# Feasibility of intraoperative indocyanine green injection to identify lymph nodes at risk of metastatic disease for early-stage lung cancer

**DOI:** 10.1016/j.xjtc.2025.05.003

**Published:** 2025-05-21

**Authors:** Desi K.M. ter Woerds, Roel L.J. Verhoeven, Stefan M. van der Heide, Erik H.J.G. Aarntzen, Monika G. Looijen-Salamon, Shoko Vos, Ad F.T.M. Verhagen, Erik H.F.M. van der Heijden

**Affiliations:** a Department of Pulmonary Diseases, Radboud University Medical Center, Nijmegen, The Netherlands; b Department of Cardiothoracic Surgery, Radboud University Medical Center, Nijmegen, The Netherlands; c Department of Medical Imaging, Radboud University Medical Center, Nijmegen, The Netherlands; d Department of Nuclear Medicine and Molecular Imaging, University Medical Center Groningen, Groningen, The Netherlands; e Department of Pathology, Radboud University Medical Center, Nijmegen, The Netherlands

**Keywords:** thoracic surgery, indocyanine green, near-infrared imaging, sentinel lymph node procedure, lymph node metastases, pathological analysis

## Abstract

**Objectives:**

Despite surgical resection and mediastinal lymph node dissection, 34% of patients with stage IB to IIB non–small cell lung cancer demonstrate recurrence within 3.5 years, indicating disease dissemination undetected by current staging methods. Although 23.1% of patients with clinical N0 disease are upstaged to pN1 or pN2 disease after surgery, occult disease may still be present in patients with pN0 disease. The aim of this research was to explore implementation of a sentinel lymph node (SLN) procedure to identify lymph nodes at risk and determine its effect on staging.

**Methods:**

In this single-center prospective study, patients with cN0 disease with resectable non–small cell lung cancer underwent intraoperative indocyanine green injection followed by the identification of up to 3 SLNs with near-infrared imaging. All lymph nodes were analyzed by conventional hematoxylin and eosin staining and the SLNs were additionally analyzed by serial sectioning and cytokeratin staining to detect tumor cells that may be missed by conventional analysis.

**Results:**

SLNs were successfully identified in all 48 patients (100%). In 3 cases, injections were incorrectly positioned, possibly leading to incorrect SLN identification. Eight patients were diagnosed with pN1 or pN2 disease postoperatively; all were detected by conventional pathological assessment. Analysis of correctly performed SLN procedures showed negative SLNs were 100% indicative of absence of metastatic spread downstream. In 2 patients, serial sectioning and cytokeratin staining of the SLN revealed isolated tumor cells in 1 N1 node.

**Conclusions:**

An intraoperative SLN procedure using indocyanine green is feasible with an identification rate of 100%. A negative SLN was an indicator for absence of metastases in lymph nodes downstream.

**Figure:**
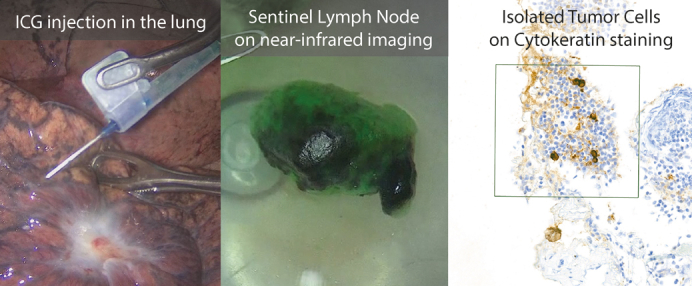
ICG injection translates to sentinel lymph nodes where isolated tumor cells were found.

**Central Message**

An intraoperative SLN procedure using ICG in patients with early-stage NSCLC is feasible but did not lead to clinical upstaging. Negative SLN is an indicator for absence of metastatic spread.

**Perspective**

The recurrence rate of 34% in patients with stage IB to IIB lung cancer within 3.5 years after intended curative resection suggests undetected dissemination. An intraoperative SLN procedure may allow for more accurate staging. SLNs were identified in 100% of patients after ICG injection into or immediately around the tumor during the operation. Negative SLNs indicate absence of metastatic spread.

The preferred treatment for early-stage non–small cell lung cancer (NSCLC) is surgical resection with complete mediastinal lymph node dissection.^1^ However, despite curative intent, recent data show a 34% recurrence rate within 3.5 years for patients with stage IB through IIB NSCLC,^2^^,^^3^ which may indicate hematogenous and or lymphatic dissemination that remained undetected by preoperative staging. In the absence of distant metastases, lymph node staging is currently based on contrast-enhanced computed tomography and 2′-deoxy-2’-[18F]fluorodeoxyglucose positron-emission tomography imaging, followed by systematic endobronchial ultrasound (EBUS). Accurate assessment of lymph node involvement is essential for effective treatment planning and prognosis. Alarmingly, we and others report that despite systematic staging in accordance with the guideline, up to 23.1% of patients with clinical stage N0 disease are upstaged to pN1 or pN2 disease after surgery.^4^, ^5^, ^6^ Conventional diagnostic techniques that are based on hematoxylin and eosin (HE) staining detect macrometastatic disease, but are postulated to overlook micrometastatic disease. A systematic review by Sun and colleagues^7^ reported that micrometastases were found in 9.3% to 62.5% of patients when immunohistochemical (IHC) and/or polymerase chain reaction techniques were used. This highlights the limitations of current staging practices and the possibility of improving techniques to detect lymphatic spread in NSCLC.

A procedure to find the sentinel lymph nodes (SLNs) has become routine clinical practice in other cancer types, like breast cancer and melanoma.^8^^,^^9^ The SLNs are defined as the first lymph nodes with an individual lymphatic drainage from the tumor lesion, making it a reference point for assessing locoregional lymphatic dissemination. The SLNs can be identified by administration of a tracer via an intra- and/or peritumoral injection that drains via the lymphatic system and accumulates in lymph nodes over time.^10^

SLN procedures have been explored in lung cancer using various techniques, including an injection with radiolabeled tracers combined with a gamma probe in a 2-day protocol, which has logistical challenges.^11^, ^12^, ^13^ Ideally, the SLN procedure would be performed during surgery to allow for immediate identification and additional pathological evaluation of lymph nodes most at risk. Near-infrared (NIR) fluorescent tracers, specifically indocyanine green (ICG), show promise toward achieving this goal. Although this has been studied by multiple groups, a procedure to identify the SLNs using ICG has not yet been widely implemented in clinical practice.^14^^,^^15^ In this study we aim to evaluate the feasibility of SLN identification using ICG injection and NIR imaging during lung surgery in patients with early-stage NSCLC and determine if more metastases could be identified through additional pathological work-up of the SLN identified by intraoperative ICG.

## Methods

### Study Design and Patient Selection

In a prospective, single-center study, the feasibility of an intraoperative SLNs procedure in patients with resectable NSCLC was assessed. Patients with cN0 disease with a tumor size of 10 to 50 mm referred for lung resection with complete mediastinal lymph node dissection were eligible for inclusion. Exclusion criteria were previous treatment for lung cancer (neoadjuvant treatment or other) or known allergy to iodinated contrast agents or ICG. The study was approved by the local Medical Research Ethics Committee (reference No. 2022-13640; approved November 29, 2022) and followed the ethical principles of the Helsinki Declaration (clinicaltrials.gov identifier NCT05555199).^16^ All patients provided written informed consent for publication of anonymized study data before participation. Enrollment took place between December 2022 and August 2024. Procedural follow-up of at least 2 weeks was available from all patients; clinical follow-up will continue for at least 5 years after study participation. Patients could be injected with ICG either via navigation bronchoscopy directly preceding surgery or transpleurally during surgery. In our surgical practice, lobe-specific systematic node dissection is always performed to the best of our abilities, according to the European Society of Thoracic Surgeons guidelines.^17^^,^^18^

### Navigation Bronchoscopy Procedure to Perform ICG Injection

When tumor identification through a surgical approach was deemed difficult or impossible, an endobronchial approach was performed when logistically feasible. After full sedation of the patient, a bronchoscope with an extended working channel (Medtronic EWC) and radial EBUS (rEBUS), or an ultrathin bronchoscope (UTB) was inserted to attempt navigation to the lesion, under fluoroscopy guidance. When the lesion was visualized by either the UTB or rEBUS, a 21G Periview Flex (Olympus Corporation) was inserted to inject 1.0 mL ICG in multiple intra- and/or peritumoral depots to ensure even distribution in or around the lesion. After ICG injection, the surgery continued as mentioned below. Ventilation of both lungs proceeded until collapse of the lung during surgery.

### Transpleural ICG Injection, Surgery, and SLN Identification

**Figure 1:**
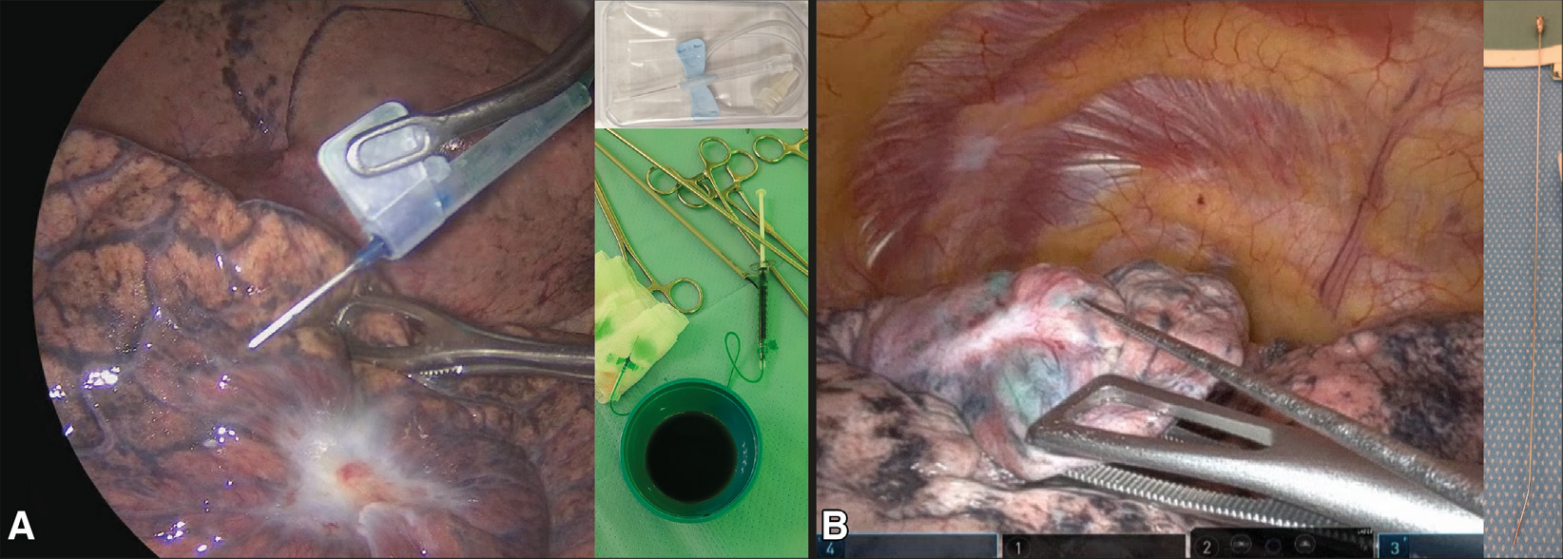
A butterfly needle (Sol-care) (A) and DeFlux needle (Link Medical Products Pty Ltd) (B) can both be used for transpleural injections depending on surgeon preference and the angle at which the lesion is located from the trocar.

Patients were fully sedated using standard general anesthesia and single-lung ventilation was achieved using a double-lumen endotracheal tube. Surgery was performed via robotic video-assisted thoracoscopic surgery ([R-]VATS) (either uniportal or multiportal) or thoracotomy. Following lung isolation, 25 mg ICG powder (Verdye; Diagnostic Green GmbH) was reconstituted in 25 mL sterile water. A syringe of 1 mL was filled and connected to a 23G DeFlux Needle (Link Medical Products Pty Ltd) or a 23G butterfly needle (Sol-care), depending on surgeon preference. See Figure 1 for illustrated examples. An attempt to inject 1.0 mL ICG evenly divided over up to 4 depots in the surrounding quadrants of the tumor was performed to cover all possible drainage patterns. If spillage outside of the injection location occurred, this was aspirated before ventilating the lung for 3 minutes. The optimal dose of ICG and ventilation time was taken from the dose-escalation study by Gilmore and colleagues.^15^ Surgery continued as per routine clinical practice, removing the tumor and performing a complete mediastinal lymph node dissection. Lymph nodes were collected in separate containers.

After removing all lymph nodes, immediate ex vivo NIR imaging by VATS optics (Karl Storz) or by R-VATS optics (Intuitive Surgical) was conducted in the operating room to determine the subjective signal intensity of ICG uptake in the lymph nodes. Up to 3 most fluorescent lymph nodes were considered SLNs and were processed as SLNs by the pathology department (see below).

### Pathological Examination

Non-SLN lymph nodes were assessed by 1 HE-stained slide, as per routine clinical practice. The SLNs were subjected to serial sectioning and HE staining on 5 levels in total. When no tumor cells were detected in the HE slides, immunohistochemical staining of the lymph nodes was performed on all 5 levels using the cytokeratin (CK) AE1/AE3 clone antibody (DAKO Agilent), provided as ready-to-use. Staining was carried out on the fully automated DAKO Omnis immunostainer, using EnVision FLEX Target Retrieval Solution, high pH. Detection was achieved with the EnVision FLEX Horseradish Peroxidase system (DAKO Agilent). Metastatic disease was categorized as macrometastasis (a group of tumor cells >2.0 mm), micrometastasis (a group of tumor cells between 0.2 and 2.0 mm), and isolated tumor cells (ITC) (single tumor cells or cell clusters <0.2 mm).^2^

### Statistical Analysis

Data were analyzed using descriptive statistics to summarize patient demographics, tumor characteristics, injection parameters, and pathological outcome. The number of patients with lymph node metastases identified only through serial sectioning and/or CK staining through this procedure was determined by 2 specialized pulmonary pathologists (S.V. and M.L.). Difficult cases were discussed together to reach consensus. To eliminate bias in determining whether metastases were found only by additional efforts (serial sectioning and CK staining) or could also have been detected by conventional methods (1 HE-stained slide), we defined that the middle section (level 3 out of 5) should contain metastases visible by HE staining. The number needed to detect (NND) more metastasis than through 1 conventional HE slide and the NND 1 patient with clinically relevant upstaging was calculated using available formulas.^19^

## Results

A total of 50 patients were included in this feasibility study. Two patients were excluded from analysis. One due to the lung being adherent to the thoracic wall resulting in the loss of anatomical reference after detachment, which precluded accurate ICG injection. A second patient was excluded because ICG was intentionally injected intravenously to visualize segmental borders before transection leading to ICG accumulation in all lymph nodes, which compromised assessment of drainage. Patient, tumor, and surgical characteristics of the 48 analyzed patients are summarized in Table 1. Median follow-up time was 288 days (range, 14-805 days).

**Table 1:** Patients, lesion, and surgical characteristics

Characteristic	Frequency
Patient characteristics	
Age (y)	67 (62-72)
Gender	
Male	28 (58.3)
Female	20 (41.7)
Smoking	
Never	5 (10.4)
Former	31 (64.6)
Current	12 (25.0)
BMI	26 (23-28)
FEV1	91 (81-105)
DLCO	89 (71-102)
Lesion characteristic	
Lesion size on CT (mm)	26 (18-34)
Lobe	
Upper	32 (66.7)
Middle	0
Lower	16 (33.3)
Lesion type	
Solid	26 (54.2)
Part-solid	4 (8.3)
GGO	9 (18.8)
Cystic	9 (18.8)
Preprocedural N-stage∗	
cN0	48 (100)
Surgery	
Surgery type	
VATS	31 (64.6)
Robotic-VATS	11 (22.9)
Thoracotomy	6 (12.5)
Extent of resection	
Lobectomy	47 (97.9)
Segmentectomy	1 (2.1)
Surgery duration (hh:mm)	03:16 (02:44-03:43)

Values are presented as interquartile range or n (%). *BMI*, Body mass index; *FEV1*, forced expiratory volume in the first second; *DLCO*, diffusing capacity of the lungs for carbon monoxide; *CT*, computed tomography; *GGO*, ground glass opacity; *VATS*, video-assisted thoracoscopic surgery.

∗ After endobronchial ultrasound.

### ICG Injection and SLN Procedure

**Figure 2:**
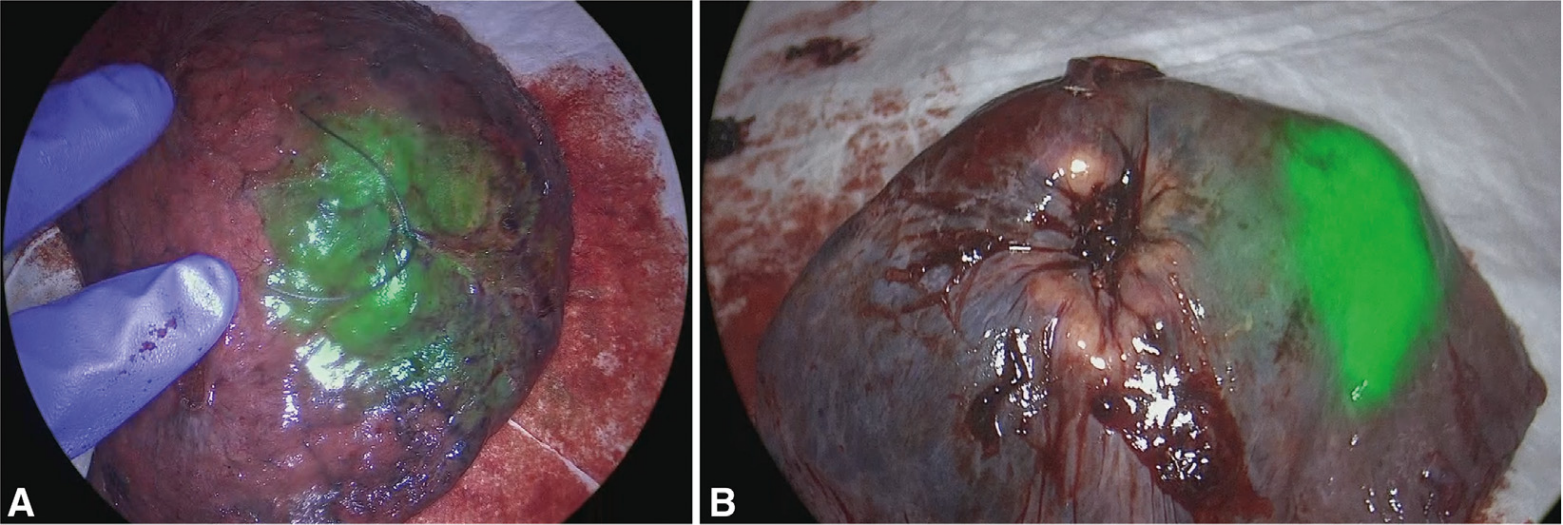
Two examples of transpleurally injected indocyanine *green* visible on the lung surface directly after resection. A, In the correct location because the lesion is located at the suture. B, In the incorrect location, too far away from the tumor (which is located at the area of pleural retraction).

Three patients received transbronchial ICG injection(s) (median, 1 depot; range, 1-3 depots) of 1.0 mL (range, 0.8-1.0 mL). The other 45 patients received transpleural ICG injection during surgery with a median of 4 depots (range, 3-5 depots) totaling 1.0 mL (range, 1.0-1.2 mL). Successful injection localization was achieved in all patients injected by navigation bronchoscopy (by rEBUS or UTB visualization) and in 42 out of 45 patients who underwent transpleural injections by fluorescent imaging directly after resection (Figure 2). In the 3 cases where the tumor was not visible or palpable, injection success could only be determined after resection by visualization of the tumor or palpation in combination with fluorescent imaging.

SLNs were found in all patients (100%) following ICG injection. The median number of removed lymph nodes per patient was 7 (range, 4-12) and a median of 3 SLNs were allocated per patient (range, 1-6). Intensity measurement of the fluorescence signal was not possible using the equipment available, and therefore comparison of inter-patient variability in NIR signal was not feasible. See Table 2 for all ICG injection and SLN characteristics.

**Table 2:** Surgical and injection characteristics

Characteristic	Frequency
Surgery	
Type of procedure for injection	
Transbronchial∗	3 (6.2)
Transpleural	45 (93.8)
Lesion locatable for transpleural injection†	35 (77.8)
ICG injection	
Transbronchial	
No. of depots	1 (1-1)
Total injected volume (mL)	1.0 (0.75-1.0)
Endobronchial leakage of ICG	1 (33.3)
Injection success	3 (100)
Transpleural	
Needle used for injection	
Butterfly needle 23G‡	14 (31.1)
DeFlux needle 23G§	29 (64.5)
Conventional needle 21G	2 (4.4)
No. of depots	4 (4-4)
Total injected volume (mL)	1.0 (1.0-1.0)
Transpleural ICG leakage	21 (46.7)
Injection success	42 (93.3)
SLN procedure	
No. of removed lymph nodes per patient	7 (6-10)
No. of fluorescent lymph nodes per patient	3 (2-5)
SLN identification	48 (100)
No. of SLNs per patient	3 (2-3)
NIR optics for fluorescence detection	
Da Vinci Firefly||	2 (4.2)
Karl Storz NIR	46 (95.8)
Pathology outcome	
Histology type	
AC	37 (77.1)
SCC	10 (20.8)
AC-NEC	1 (2.1)
N-stage	
pN0	38 (79.2)
pN0 (i+), ITC	2 (4.2)
pN1	5 (10.4)
pN2	3 (6.2)

Values are presented as interquartile range or n (%). *ICG*, Indocyanine green; *SLN*, sentinel lymph node; *NIR*, near-infrared; *AC*, adenocarcinoma; *SCC*, squamous cell carcinoma; *NEC*, neuroendocrine carcinoma; *ITC*, isolated tumor cells.

∗ By navigation bronchoscopy.

† Only scored in patients who underwent a transpleural ICG injection.

‡ Sol-care.

§ Link Medical Products Pty Ltd.

|| Intuitive Surgical.

### Monochromatic and Overlay Setting of NIR Imaging

**Figure 3:**
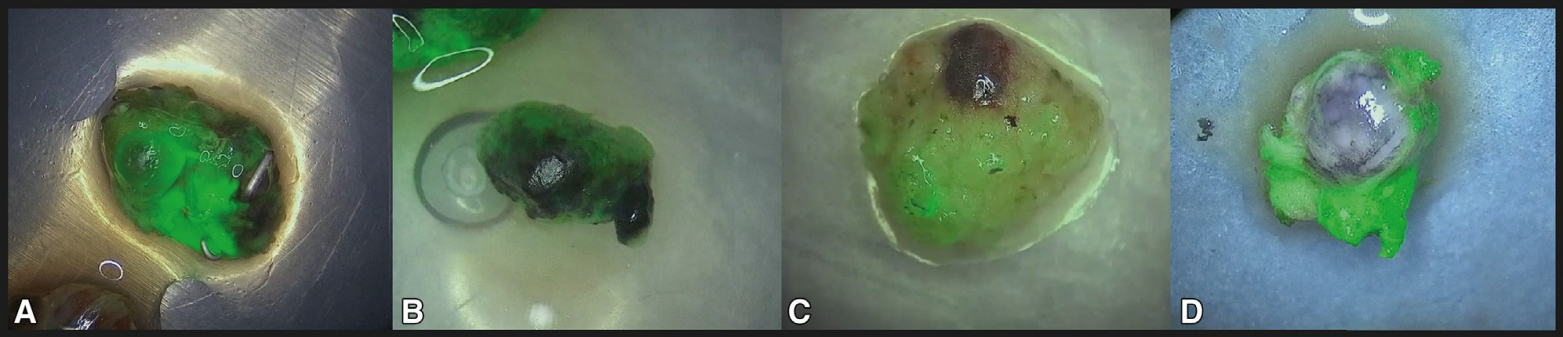
Two examples of indocyanine *green* fluorescence within lymph nodes (A and B) and 2 examples of fluorescent signal found in the fatty tissue surrounding the lymph node (C and D).

**Figure 4:**
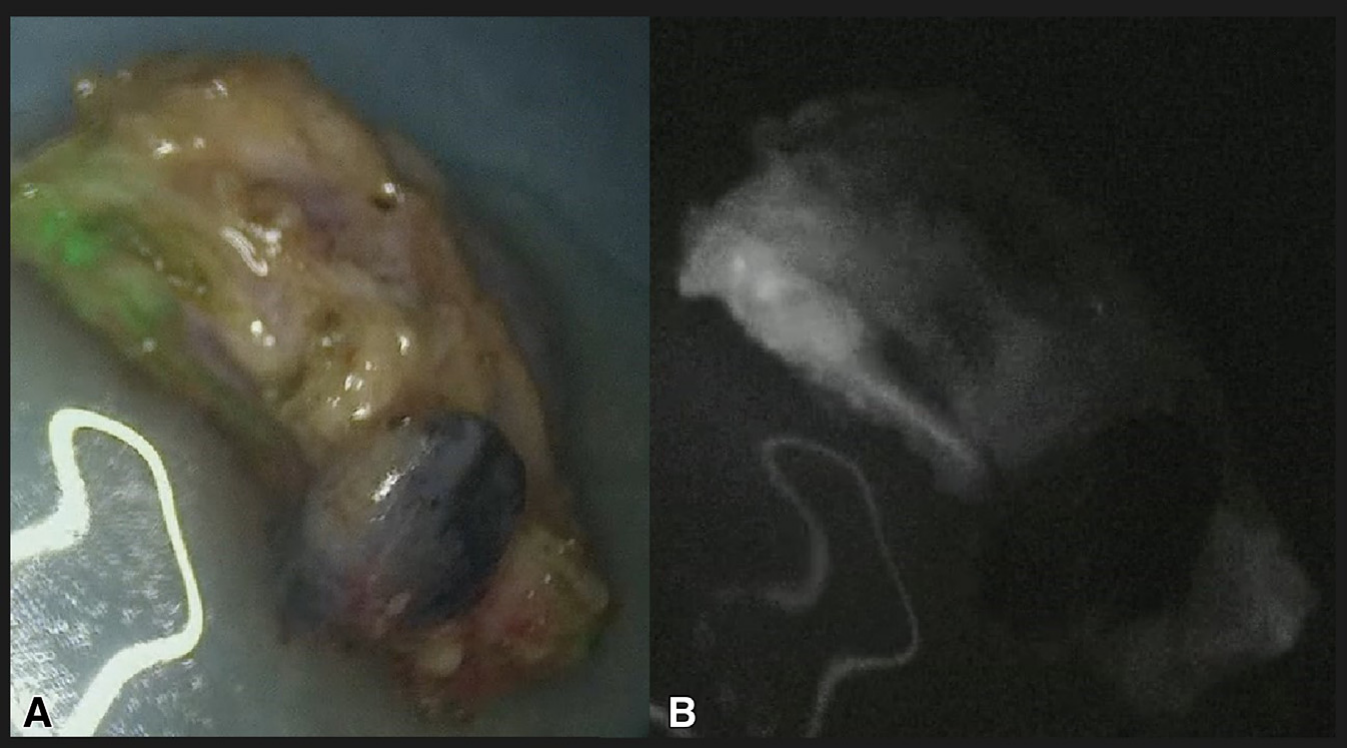
An example of a lymph node where monochromatic view with only fluorescent signal (B) revealed a complete lack of fluorescence signal in the lymph node itself. This was not possible in the image with only pseudocolor overlay of the fluorescent signal (A).

Visualization of ICG in the lymph nodes revealed not only uptake by the lymph node itself, but also in the surrounding fatty or connective tissue that is frequently resected with the lymph node. Lymph nodes were initially only assessed by NIR imaging in the overlay setting, which is a combined image of the green pseudocolor fluorescent signal on top of the color image produced by white light.^20^ Because the green pseudocolor is projected on top of the normal color image, it can be difficult to determine if the fluorescent signal is present in the lymph node or in the surrounding fatty tissue. This may hamper the identification of ICG in nodes as illustrated in Figure 3. Fortunately, we noticed that this could be partly solved by using the monochromatic image setting that only displays the NIR signal (see Figure 4).

### Clinical Consequences of Pathological Analysis in SLN

All patients included had a clinical N0 status after guideline-concordant workup and staging. After surgery, 40 out of 48 patients were diagnosed with pN0 disease (83.3%) of whom 2 patients were found to have ITCs (pN0[i+]) (Table 3). Eight patients were diagnosed with pN1 or pN2 disease postoperatively (16.7%) (Table 3). The ICG injection was inaccurate in 2 of the 8 patients with pN + disease. See Figure 2 for an example of (in)accurate injections.

**Table 3:** Detailed information on all 10 patients in whom tumor cells were ultimately found in the lymph nodes

Patient no.	Tumor location	Accurate injection	SLN stations based on ICG uptake		Other nodal regions sampled and evaluated by NIR imaging		Classification of metastatic disease		
			With metastatic disease	Without metastatic disease	With metastatic disease	Without metastatic disease	Macro-metastasis	Micro-metastases	ITC
1	RLL	Yes		9 + 10 + 11		2 + 4 + 7 + 9 + 10 + 11	Peribronchial∗		
2	RUL	Yes	10 + 11		7	2 + 4 + 11	7† + 10 + 11		
3	LLL	Yes	10	7		5 + 9 + 10 + 11	10 + peribronchial∗		
4	RUL	Yes	4 + 10	7		2 + 4 + 10 + 11	10	4	
5	RUL	Yes	10	4		2 + 4 + 7 + 10 + 11		10‡	
6	RUL	Yes	11 + 10 + 7			2 + 4 + 10	11 + peribronchial∗	10	7
7	RUL	Yes	11	7 + 10		2 + 4 + 10 + 11 + 12			11
8	LLL	Yes	11	10		5 + 7 + 9 + 10			11
9	LUL	No		10 + 11	3 + 5	7 + 10 + 11	3 + 5		
10	RLL	No	11		11	4 + 10	11		

Some lymph node regions contained multiple lymph nodes with and without (sufficient) ICG uptake, and were therefore classified as both SLN and other lymph nodes based on NIR signal. Macrometastases were classified as metastases >2.0 mm, micrometastases are metastases that measured between 0.2 and 2.0 mm, and ITC are groups of cells <0.2 mm.

*SLN*, Sentinel lymph node; *ICG*, indocyanine green; *NIR*, near-infrared; *ITC*, isolated tumor cells; *RLL*, right lower lobe; *RUL*, right upper lobe; *LLL*, left lower lobe; *LUL*, left upper lobe.

∗ Peribronchial lymph nodes were not assessed by NIR imaging.

† Station 7 was fluorescent, but less so compared with 2 lymph nodes from station 10 and 1 from station 11 and was therefore not classified as an SLN.

‡ A total of 5 lymph nodes in region 10 were removed, of which 2 were SLNs. Micrometastases were found in the SLN only.

In 1 of the remaining 9 patients with pN + disease with accurate ICG injection, both the SLNs and a lymph node downstream were involved (Patient 2) (Table 3). In the remaining 5 patients, no metastases were found in lymph nodes downstream from the identified positive SLN. In 3 out of these 5 patients, metastases were also found upstream, in the peribronchial lymph nodes (region 12-14), which were located deep in the lobectomy specimen and therefore not assessable by NIR imaging during surgery and not classified as SLNs. Details on detected metastases in SLNs or other lymph nodes can be found in Table 3.

In all patients with a pathology-negative SLN and an accurate ICG injection, no metastatic disease was detected in any of the lymph nodes located downstream. No correlations were found between metastatic disease found after surgery and pre- or intraprocedural patients or lesion characteristics.

### Value of Serial Sectioning and CK Staining for Detection of Metastatic Disease

**Figure E1:**
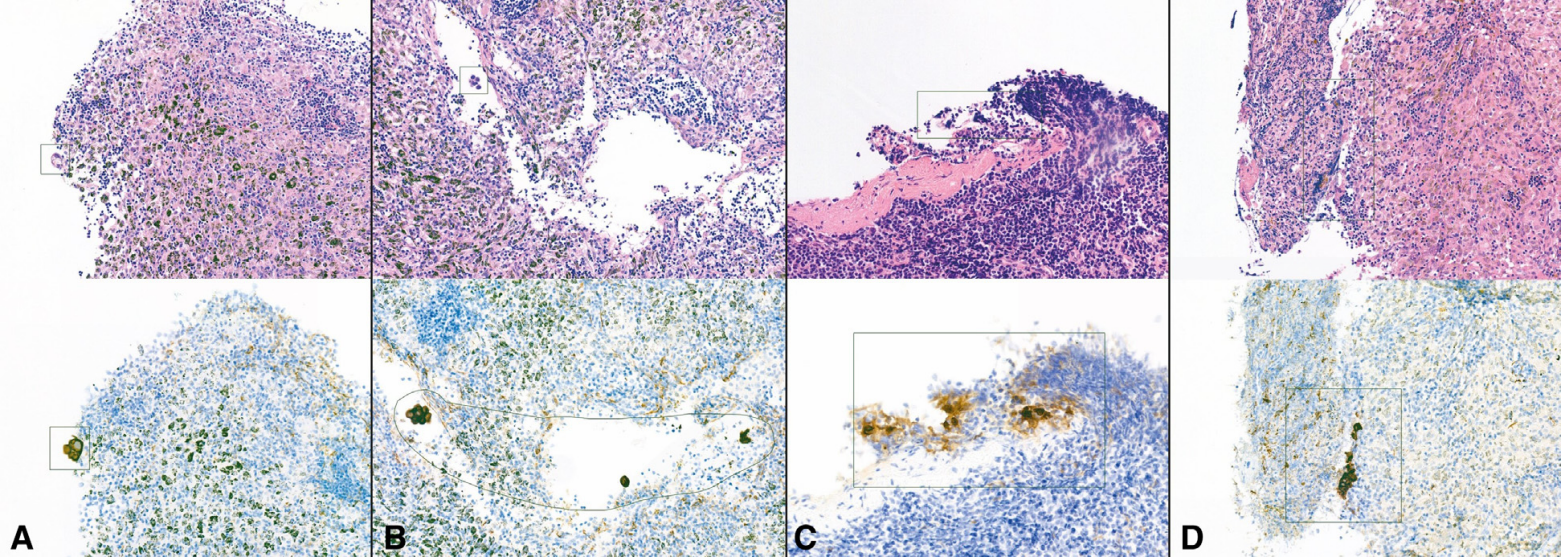
Annotated images of the hematoxylin-and-eosin stained and cytokeratin stained slides of lymph nodes where isolated tumor cells (ITCs) were found. A and B, Patient with ITCs on the border and in lymph node station 11. C, Patient with ITC on the border of station 11. D, Patient with pN1 disease in stations 10 and 11, and ITC in station 7 (depicted).

Serial sectioning and CK staining of the SLN identified no micro- or macrometastases that would not likely have been found using the conventional workup with a single-level HE staining. These additional efforts did identify ITC in the SLNs of 3 patients of whom 2 received a N0(i+) stage and 1 was already diagnosed with pN1 disease. Clinical interpretation was complicated because the ITC were mostly found on the edges of the lymph node (see Figure E1). Because ITCs are currently not a reason for upstaging, the NND to upstage 1 patient by this technique could not be calculated. The NND 1 patient with ITC (N0[i+] stage) was 24 patients.

### Safety and Adverse Events

No adverse events related to the study procedure were observed apart from 1 patient who had a small parenchymal bleed caused by the trocar still in situ during the ventilation after ICG injection. No intervention was needed because this lobe was to be removed anyway. Transbronchial ICG injection per navigation bronchoscopy prolonged surgery duration by approximately 25 minutes, and approximately 5 minutes were added to perform transpleural injections.

## Discussion

This study demonstrates that SLN identification by ICG injection and NIR imaging during surgery in patients with early-stage NSCLC is feasible and allows for targeted evaluation by pathology. A total of 48 patients with cN0 disease were included, of whom 8 were diagnosed with pN + disease. Of these 8 patients, 2 had inaccurately placed ICG injections. In patients with a successful peritumoral ICG injection and pathology-negative SLNs, no downstream metastatic spread was found. Regarding to pathological workup, serial section and CK staining did reveal 3 cases of ITC but did not reveal any macro- or micrometastatic disease that would not have been found by routine histopathological evaluation and thus did not influence staging outcome. Based on these results, the NND 1 patient with ITC to receive a pN0(i+) instead of pN0 stage is 24 patients. But, as per current guidelines and the new ninth edition of the TNM staging manual, ITC does not affect pathological staging nor the eligibility for adjuvant treatments in these patients.^21^

In contrast to lung cancer, the identification of SLN and detailed analysis thereof in the workup of other cancer types has become a routine clinical procedure. This widely accepted SLN methodology has been shown to improve staging accuracy and decrease recurrence through eligibility to tailored treatments.^22^^,^^23^ The accuracy of SLN detection in lung cancer is still unknown. One key factor is whether the injection of ICG can always be accurately placed within the target area. We have found that when an accurate injection is not possible, there is no value in finding the lymph node with the most ICG uptake. It is recommended to select cases where ICG injection will be possible based on preoperative imaging and this could aid in the standardization of the technique. A second limiting factor is the lack of quantitative measurement of fluorescence to determine which lymph node contains the highest concentration of ICG and if this node truly represents the first site of metastasis in all cases. A complete lymphadenectomy remains necessary to determine the 3 most fluorescent lymph nodes, which is inherently a subjective manner. Compared with other cancers, the procedure performed during lung surgery cannot determine which lymph nodes should be resected and which can remain in situ when no metastases are found. The presented technique to find the SLNs is therefore only applicable to improve staging of lymph nodes by focused pathological and IHC techniques.

### Finding Micrometastases or ITC by IHC

A systematic review by Sun and colleagues^7^ reported that more micrometastases are found when IHC and polymerase chain reaction techniques are used on top of conventional HE staining. These techniques are time-consuming and in our hands serial sectioning and CK staining of identified SLNs did not lead to clinically relevant changes in staging outcome because only ITCs were (likely additionally) detected. Furthermore, as illustrated in Figure E1, ITCs were mostly found on the edges of the lymph node instead of inside the lymph node and CK-AE1/AE3 generally shows background staining in, for instance, interstitial reticulum cells that could also be interpreted as contamination.^24^^,^^25^ It is important to accurately diagnose these metastases because studies show that patients with either micrometastases and/or ITC may have poorer survival than patients without metastases.^2^^,^^26^^,^^27^ Unfortunately, most meta-analysis or systematic reviews group patients with micrometastases and ITC, making it impossible to determine the influence of diagnosing only ITC on the prognosis of a patient. With the current guidelines, the presence of ITC (<0.2 mm, pN0[i+]) does not change nodal stage, whereas the identification of micrometastases (0.2-2.0 mm, pN+) does translate into upstaging, making patients potentially eligible for additional treatment.^28^ Future studies will need to determine whether patients diagnosed with ITC have a poorer prognosis compared with micro- or macrometastases or patients without metastases and whether or not they should also be eligible for adjuvant treatment, potentially contributing to more personalized treatment.

### SLN Identification Rate

Using the presented study protocol, SLNs were identified in 100% of patients when depots were accurately placed. Most other studies report identification rates of 75% to 82%.^29^, ^30^, ^31^, ^32^ Digesu and colleagues^33^ even reported a 100% identification rate in selected cases. As Phillips and colleagues^32^ and Gilmore and colleagues^31^ both show in their dose escalation studies, several parameters and protocol variations may be of influence in achieving adequate SLN identification. It can be concluded by their and our results that ventilation of the lung after injection and an ICG dose of at least 1 mg are essential for SLN identification.^15^^,^^29^^,^^31^^,^^32^ A very recent study by Stasiak and colleagues^34^ reported an identification rate of 90.6% in 6 patients after injection of 2.5 mg ICG in 1 mL. In line with our findings, they also found that when the SLNs are clean, all lymph nodes downstream are free of tumor cells as well. They also performed injections transpleurally as well as endobronchially and noticed a slightly lower SLN identification rate with the transpleural approach compared with the endobronchial approach.^34^ Unfortunately, our endobronchial cohort is too small (n = 3) to compare NIR uptake in the lymph nodes.

Although we have only investigated the injection of ICG, tracers with radioactive or magnetic properties or even visible dye have also been explored in the past. These agents have generally been studied more extensively but have not demonstrated consistent effectiveness or require complex logistics. ICG remains relatively underexplored in this context.^35^^,^^36^ However, the heterogeneity in study outcomes by us and others show that more work is needed to better understand which methodology would provide for a universally successful approach.

### Finding the Lymph Nodes Most at Risk

Some surgeons have explored and implemented lobe-specific lymph node dissection in an attempt to find the lymph nodes most at risk.^37^ We noticed in our cohorts of patients with both pN0 and pN+ disease that when multiple lymph nodes from a single region were resected, some lymph nodes contained ICG, whereas others contained no ICG at all. This suggests that not all lymph nodes in a region are draining lymph fluid from the lung tumor similarly. Although the identification of lobe-specific lymph nodes could minimize extra pathological efforts somewhat, SLN identification would theoretically have more potential to identify the lymph nodes most at risk. And although we found a 100% SLN identification rate in correctly placed injections, additional pathological efforts by CK staining and additional sectioning did not seem to detect more metastases. Based on this observation, we expect that lobe-specific lymph node dissection would also not have revealed more metastases in this cohort.

### Strengths, Limitations, and Future Considerations

A prominent limitation of our ICG-based SLN procedure is the risk of spillage into the thoracic space after injection, which could possibly result in more fluorescence appearing in the fat surrounding the lymph nodes than in the lymph node itself. This can make differentiation between true nodal uptake and adjacent background signal challenging. As we illustrated, the overlay images were occasionally misleading because the over effect caused ICG to appear as originating from the node when it was in fact located in the fatty tissue surrounding the lymph node. The monochromatic view could partly alleviate the problem because it provided more depth to the NIR signal, but it was not helpful enough when the lymph node was fully embedded in the fatty tissue. This is largely attributed to the subjective nature of the technique and could lead to false-positive or false-negative findings. A second limitation of this technique is the inability to evaluate intrapulmonary lymph nodes by NIR imaging because these are only found after fixation of the removed lung tissue and are not available at the time of NIR evaluation. These lymph nodes are also embedded close to the fluorescent lung tumor, which makes evaluation without resection difficult due to the overshine of the ICG depots in the resected lung. The presented technique is therefore only applicable to improve staging of lymph nodes that are separately removed by a surgeon. Although not performed by us, removal techniques of the more peripheral lymph nodes in station 12 to 14, as demonstrated by Raymond and colleagues,^38^ could perhaps be utilized in future to be able to evaluate all lymph nodes with NIR imaging.

This study was performed in a single center, which might have affected the reproducibility of surgical techniques as well as pathological analysis. Additionally, the sample size of the study was small and does not allow for statistical analysis regarding metastatic disease. Moreover, this study only included patients with cN0 staging and it is unclear if treatment of lymph node metastases would influence the drainage of lymph fluid to the lymph nodes and whether a patient could still benefit from additional staging efforts. Considering the rise of neoadjuvant treatment,^39^^,^^40^ we believe these patients should be included in future studies on intraoperative SLN procedures to find possible limitations or advantages and define clear inclusion criteria to maximize procedure outcomes.

## Conclusions

Finally, at the time of writing, the longest follow-up period in our cohort is 1.5 years. This is insufficient to draw final conclusions about the added accuracy of staging, specifically with respect to disease recurrence. A longer follow-up period and larger population will be needed and will be collected to determine if the recurrence rate in the group of patients with pN0 is different from other patients with pN0 disease in our center and whether implementation of this technique would add value to patient outcome.

## Conflict of Interest Statement

Drs Verhoeven and van der Heijden report unrestricted research grants to the department from AstraZeneca Oncology, Pentax Medical, Philips, Johnson & Johnson, Intuitive, KWF (national cancer fund), and IMAGIO (Innovative Health Initiative—EU fund); consulting fees to the department from Johnson & Johnson, Intuitive, and NLC; travel arrangements from Johnson & Johnson and Intuitive; and in-kind support and other loan of equipment for performing a clinical study from Philips Medical, Intuitive, and Pentax Medical. Dr Verhoeven is chair of the Dutch Society of Technical Physicians. Dr van der Heijden reports speaker fees to the department from Janssen-Cilag, Siemens, Pentax, Ethicon, Astra Zeneca, Intuitive, and Philips; travel arrangements from Siemens; and is an executive board member of the European Association for Bronchology and Interventional Pulmonology. All other authors reported no conflicts of interest.

The *Journal* policy requires editors and reviewers to disclose conflicts of interest and to decline handling or reviewing manuscripts for which they may have a conflict of interest. The editors and reviewers of this article have no conflicts of interest.
